# Strategies for Safeguarding High-Risk Pregnancies From Preterm Birth: A Narrative Review

**DOI:** 10.7759/cureus.55737

**Published:** 2024-03-07

**Authors:** Hussam A Al Hussaini, Rahaf K Almughathawi, Renad M Alsaedi, Ghadah A Aljateli, Ghofran Saleem M Alhejaili, Munira A Aldossari, Abdullah S Almunyif, Raghad K Almarshud

**Affiliations:** 1 Obstetrics and Gynecology, Maternity and Children Hospital Tabuk, Tabuk, SAU; 2 Obstetrics and Gynecology, Alrayan Medical Colleges, Madina, SAU; 3 Obstetrics and Gynecology, Unaizah College of Medicine and Medical Sciences, Qassim University, Unaizah, SAU; 4 General Medicine, Maternity and Children Hospital Madina, Madina, SAU; 5 Obstetrics and Gynecology, King Saud bin Abdulaziz University for Health Sciences, Riyadh, SAU; 6 General Practice, Ministry of Health Holdings, Riyadh, SAU

**Keywords:** high risk, pregnancy, premature birth, preterm birth, prevention

## Abstract

Preterm birth is the delivery of a live fetus before the 37th week of gestation. Preterm birth may stem from various factors, including premature rupture of membranes, spontaneous preterm labor, or medically induced circumstances. Premature delivery can result in serious and long-lasting difficulties even for infants who survive, as it is the leading cause of death for infants under five years old. Numerous nations have implemented initiatives to detect and track pregnant women who may give birth before their due date. Numerous therapies are available to protect these at-risk groups from the devastating effects of premature delivery, given the complex nature of preterm birth risk factors.

Among the preventive measures, prophylactic progesterone appears to hold significant promise, while cervical cerclage proves effective in cases of cervical insufficiency. Conversely, pessaries show no discernible beneficial effects in reducing the risk of preterm birth. Regular antenatal visits are imperative for frequent patient evaluation and screening for potential risk factors. Adopting a healthy lifestyle can influence the risk of developing preeclampsia, with regular physical activity, a fiber-rich diet, and smoking cessation serving to mitigate the risk of preterm birth. The efficacy of bed rest in preventing preterm birth remains inconclusive due to insufficient evidence. This study aims to explore various preventive strategies for averting premature birth in high-risk women.

## Introduction and background

Preterm birth is the delivery of a live fetus before 37 weeks of gestation or before 259 days, as defined by the World Health Organization [1]. Preterm birth can be classified according to the cause and the timing. The classification by cause is divided into three main categories: medically induced, premature rupture of membranes, and spontaneous preterm birth. Several factors, whether maternal or fetal, may require the induction of labor before full term. These factors include maternal cardiovascular issues, chronic illnesses, or obstetric complications. Additionally, the fetus may experience critical problems that exacerbate the situation, such as intrauterine growth restriction, multiple pregnancies, or other conditions that compromise fetal stability. Premature rupture of membranes can be attributed to many causes, such as African American ethnicity, infections, and uterine distention [2]. Spontaneous premature birth has several risk factors, like the extremes of weight, racial Hispanic African Americans, low socioeconomic status, smoking, and stress [3].

Three categories apply to preterm birth: very preterm (between 28 and 32 weeks of gestation), extremely preterm (less than 28 weeks of gestation), and moderate to late preterm (between 32 and 37 weeks of gestation). A study analyzing data from 103 countries and regions revealed that approximately 15% of preterm births occurred before the completion of 32 weeks of gestation. Notably, Southern Asia and sub-Saharan Africa collectively contributed to 65% of the documented cases of preterm births. This study showed that the preterm births worldwide in 2020 were 13.4 (95% CI, 12.3-15.2) million, compared to 13.8 (95% CI, 12.7-15.5) million in 2010 [4].

The outcomes and prognosis of preterm birth are dependent on the age of gestation at delivery and on the weight [2]. Although prematurity was previously defined as babies under 2.5 kg, this isn’t the case anymore, as although preterm babies tend to be smaller in comparison with full-term babies, some term babies may be small too; they are called smaller for gestational age, which can be tracked using Fenton charts for boys and for girls [5]. Preterm birth can have serious effects on the health of the newborn, not only because it is the leading cause of mortality in newborns under five years, but also because, even with survival, it can lead to serious complications such as cerebral palsy, neurological, gastrointestinal, vision, hearing, and others [1,6].

The purpose of this review is to offer a comprehensive guide for preventing preterm birth in high-risk women. It aims to briefly present predictive and diagnostic methods, serving as a valuable resource for physicians managing women at elevated risk of experiencing premature delivery.

## Review

Methods

We searched PubMed, Scopus, and Web of Science using the following terms: ("preterm birth" OR "preterm labor") AND (progesterone OR "17α-hydroxyprogesterone" OR pessary OR cerclage OR fibronectin OR homocysteine) AND (prevention OR safeguarding). The eligibility of articles was screened based on their relevance to explore the strategies that help to avoid preterm birth in high-risk pregnancies without any filters or limitations based on research design.

Women at high risk of preterm birth

Many causes can lead to preterm birth; our role is to provide effective methods for its prevention to avoid the consequences. New techniques using machine learning to predict the women at risk of having preterm birth are being developed, but they are hard to interpret [7,8].

Although UK NICE guidelines for multiple pregnancies recommend no screening in the absence of effective management [9], various tests are available for screening women at a high risk of preterm birth, including home uterine activity monitoring, digital cervical examination, ultrasound assessment of cervical length, screening for bacterial vaginosis, evaluation for fetal fibronectin, and examination of amniotic sludge. These tests can also be used for screening high-risk women with singleton pregnancy [10]. So, we need to identify the women at risk and then proceed with the best management according to each case.

It is important to know what the risk factors are that are associated with preterm birth; thus, we can predict women at risk and manage the cases properly to avoid the consequences of preterm birth. In an umbrella review [11] of 1511 primary studies discussing 170 associations, only seven of those associations had a robust association with preterm birth. This study argues with the WHO guidelines [12] of waiting for at least six months to conceive after a miscarriage; this is supported by excluding a large cohort study. Conde-Agudelo et al. (2004) [13] form this meta-analysis. With all the present evidence, this study didn’t differentiate between spontaneous and induced abortion and included studies from countries where abortion is illegal. By excluding this study, the remainder of the evidence suggested that not waiting for six months after miscarriage has better outcomes for the next pregnancy because the mother is usually more fertile and more attentive to her health during the next pregnancy. The outcomes of miscarriage (under 12 weeks) are better than later pregnancy after 12 weeks and delivery later in pregnancy due to the effect on folate reserve.

The study also showed that sleep breathing disorders such as sleep apnea could have great outcomes on pregnancy and perinatal outcomes, especially with the increased rates of obesity. Women should be screened for these problems to prevent maternal intermittent hypoxia and improve the outcomes of pregnancy, as well as maternal personality disorders, so maternal psychological screening is an important preventive measure of poor outcomes. Low gestational weight gain, previous termination of pregnancy with vacuum aspiration, and isolated single umbilical artery all showed a strong association with the risk of preterm birth.

Some risk factors showed a high suggestion of association with preterm labor; some were racial like black or African and aboriginal ethnicity, some were social as unmarried women and women who are subjected to intimate partner violence, women with severe morbidity as hemorrhagic and hepatic disorders, chronic kidney disease, bleeding in the first trimester, placenta previa, velamentous cord insertion, obstetric cholestasis, obesity (BMI >40 kg/m^2^), underweight women, maternal age equal or more than 45 years, prior surgical termination of pregnancy, history of cancer, and cervical intraepithelial neoplasia treatment [11].

**Figure 1:**
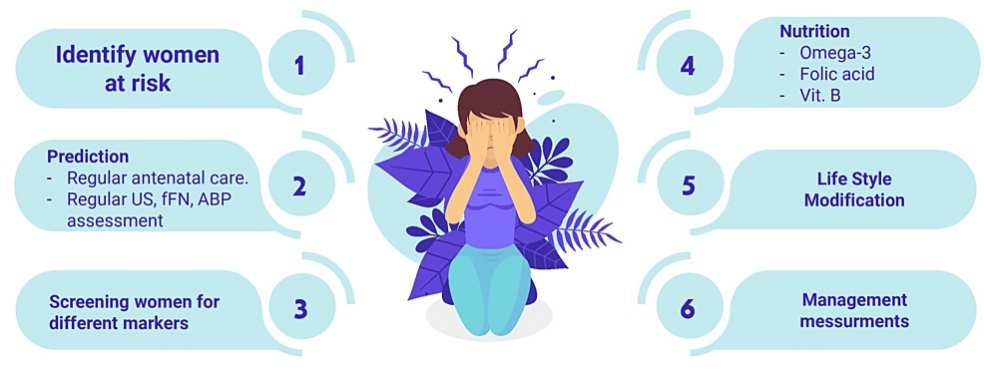
Prevention of preterm birth in high-risk women ABP: arterial blood pressure; fFN: fetal fibronectin; US: ultrasound; Vit.: vitamin Image Credit: Authors

Chorioamnionitis is an inflammatory process in the chorion, amnion, and placenta that leads to the release of cytokines. It can be symptomatic with fever, leukocytosis, tachycardia, uterine tenderness, and prelabor rupture of the membranes (PROM), or it can be asymptomatic, which is much more commonly associated with preterm birth. Chorioamnionitis can be associated with fetal inflammatory response syndrome, leading to renal, cardiopulmonary, and neurological symptoms, but it can also increase the risk of preterm birth [14]. Figure 1 shows how to prevent preterm birth in high-risk women.

Fibronectin

Fetal fibronectin (fFN), which is detectable in cervicovaginal secretions, can be used as a predictor to distinguish between women who are at high and low risk of preterm delivery. Qualitative fFN is a good negative test, enabling us to detect women with low risk [15]. The fFN test is an enhanced method for determining the probability of spontaneous preterm birth in asymptomatic women with predisposing conditions. This test determines whether these women are at a high or low risk of experiencing preterm birth before the 37th week of gestation. The likelihood of spontaneous preterm delivery significantly increases with higher fFN levels, with a fourfold increase for levels ≥50 ng/ml and a sevenfold increase for levels >200 ng/ml, compared to fFN levels below 50 ng/ml. Additionally, the quantitative fFN test aids in identifying women at immediate risk of spontaneous preterm birth, enabling the initiation of antenatal corticosteroids to facilitate lung development and prevent neonatal respiratory distress syndrome [15].

Homocysteine

Homocysteine can be used to predict preterm birth and other complications of pregnancy, especially in developed countries with fewer resources and unplanned pregnancies [16]. A defect in homocysteine metabolism can lead to its accumulation. It has been linked to some nutrient deficiencies, particularly vitamin B, mainly folic acid, so hyperhomocysteinemia has been linked to many diseases such as preeclampsia, neural tube defects, and recurrent miscarriages. In a prospective cohort study conducted in Nigeria, 24.6% of the study population showed elevated levels of homocysteine, and the risk of preterm birth in women with hyperhomocysteinemia was about twelve times higher compared to women with a normal level of homocysteine (P=0.001). It also showed a strong association with low birth weight in term children, but no effect on antenatal fetal death [16]. Administering folic acid for 12 weeks in infertile patients with a history of failed in vitro fertilization or intracytoplasmic sperm injection can significantly decrease homocysteine levels (P=0.00146); there is also an inverse relationship between vitamin D3, fertility, and homocysteine [17].

Progesterone

Progesterone can be used in multiple forms, including vaginal, oral, and 17α-hydroxyprogesterone caproate (17-OHPC). Data from three meta-analyses showed that progesterone can decrease the risk of preterm birth when compared to no treatment or placebo [18-20].

In the study by Care et al. (2022), a network meta-analysis involving women at risk of spontaneous preterm birth (defined by a history of spontaneous preterm birth or a short cervical length) indicated a low risk of preterm birth before 34 weeks with the use of vaginal progesterone and 17-OHPC [18].

The same outcomes can be seen in the Evaluating Progestogens for Preventing Preterm Birth International Collaborative (EPPPIC) study [19]. Vaginal progesterone and 17-OHPC reduced the risk of preterm birth in singleton women with a high-risk pregnancy, relative risk (0.78, 95% CI, 0.68-0.90) and (0.83, 95% CI, 0.68-1.01), respectively. Both treatments caused a decrease in the incidence of perinatal death. Vaginal progesterone can decrease the risk of ICU admission, NICU, and respiratory assistance needed; it can also decrease low and very low birth weight.

Different results are presented by Phung et al. (2022) [20]. They did a meta-analysis of three trials, all of which are of high quality, and showed that vaginal progesterone didn’t show a statistically significant decrease in the risk of preterm birth in 1,127 asymptomatic, normal mid-gestation cervical length women with a singleton pregnancy. The relative risk was 0.76 (p=0.45) for spontaneous preterm birth before 37 weeks and 0.51 (p=0.35) for spontaneous preterm birth before 34 weeks. The different results may be attributed to the difference in the population of high-risk women in 2022 [18]. They were restricted to women with a history of preterm birth and a short cervix. In the study by Phung et al. (2022) [20], they only included asymptomatic women with normal mid-gestation cervical length, and the EPPPIC study [19] included all women at high risk of preterm birth.

The guidelines from the American College of Obstetricians and Gynecologists specifically recommend the use of vaginal progesterone in cases where the cervix measures less than or equal to 25 mm. This treatment may be considered for women with a history of preterm birth or singleton pregnancy. It's important to note that the effectiveness of vaginal progesterone has been demonstrated primarily in cases with a short cervix. [21]. The FDA concluded that, based on the current evidence, prophylactic 17-OHPC, which is described for all women with prior spontaneous preterm birth history between the 20th and 37th weeks of gestation, doesn’t seem to be beneficial in any other population [21].

Pessary

Vaginal pessaries are mechanical devices with a cone-shaped apex like the fornix of the vagina, which is inserted and left inside the vagina to provide support for the vaginal walls and its apex. They are usually used to treat pelvic organ prolapse. Pessaries (like ring pessaries or space-filling pessaries such as cube pessaries) can support the female genital tract. Pessaries are compatible with sexual intercourse, have less retention risk, and are easier to remove and replace. Ring pessaries are more commonly used in clinical practice [22].

Pessaries arguably can provide support for women with a short cervix, which may cause insufficiency and spontaneous preterm birth. The pessaries are proposed to offer a noninvasive measure with no need for anesthesia in comparison with cerclage.

This Cochrane systematic review showed that pessaries had beneficial effects on preventing preterm birth and prolonging pregnancy only with women with singleton pregnancy and a short cervix less than 25 mm [23], but these results came from a single randomized trial. Goya et al. (2012) found spontaneous delivery before 34 weeks of gestation to be 6% in the pessary group and 51% in the control group (OR=0.18) [24]. The FIGO good practice recommendations have recommended not to use pessaries to decrease the frequency or improve the outcomes of preterm birth, neither in singleton pregnancy with a short cervix nor in twin pregnancy, irrespective of the cervical length [25].

Pessaries can be used to decrease the risk of spontaneous preterm birth. In a meta-analysis of 3,911 women, there was a decrease in the risk of preterm birth before 34 weeks in multiple pregnancies with a relative risk of 0.65, but it wasn’t beneficial in singleton pregnancy [26].

While a 2020 network meta-analysis debated that pessaries have no added value in preventing preterm birth at any time, either before 28, 32, or 37 weeks of gestations, or at any cervical length, neither in singleton pregnancy nor in multiple pregnancies, furthermore, pessaries shouldn’t be used in singleton women with short cervix who are taking vaginal progesterone as they may cause complications when used together [27].

Cerclage

Cerclage is a surgical procedure that was first introduced in the 1950s by Shirodkar and McDonald. It is usually indicated for women with a history of second-trimester loss of pregnancy or who have cervical insufficiency. There are three major criteria used now to indicate cerclage in singleton pregnancy: first, the history of multiple preterm births or second-trimester losses; second, that ultrasound shows a short cervix <25 mm before week 24 of gestation in a woman with a history of preterm birth; and lastly, that there is cervical dilation on physical examination before week 24 of gestation [28].

Women with cerclage are less likely to have preterm birth compared to women with no cerclage [29]. Abdominal cerclage can be used instead of vaginal cerclage, especially in women with previously unsuccessful cerclage. Transabdominal cerclage reduced the risk of preterm birth <32 weeks by 8%, while vaginal cerclage reduced it by 13% (RR=0.33) [30].

Cerclage presents itself as a viable option for preventing preterm birth, particularly in the context of multiple or twin pregnancies, by addressing cervical insufficiency. Preconception laparoscopic cervical cerclage (LCC) is a potential approach for women at a heightened risk of severe preterm birth or those with a high risk of second-trimester miscarriage. Notably, 82.60% of women who underwent LCC delivered at or after 37 weeks of gestation. Remarkably, all neonates born under these circumstances exhibited no mortality or complications. Furthermore, among those who surpassed the first trimester, there was a 100% neonatal survival rate [31].

Progesterone, cerclage, and pessary

In Care et al. (2022) [18], 40 trials with 13,310 pregnant women used data to assess the risk of preterm birth at less than 34 weeks. There was a low risk of preterm birth with vaginal progesterone (OR=0.50), Shirodkar cerclage (OR=0.06), 17-OHPC (OR=0.68), vaginal pessary (OR=0.65), and omega-3 (OR=0.30) [18].

All the interventions showed a reduced risk of preterm birth, all with moderate certainty, except for vaginal progesterone, which was of high certainty. Thirty trials showed that only progesterone reduced perinatal death (OR=0.66) with moderate certainty [18]. Only progesterone treatments reduced postnatal neonatal complications such as neonatal sepsis, necrotizing endocarditis, ICU admission, and neonatal respiratory distress syndrome [18].

Similar results were presented in the study by Jarde et al. (2017) [32], with progesterone causing the most significant reduction in the risk of preterm birth before 32, 34, and 37 weeks. The study presented very low-quality evidence that cerclage may increase the gestational age at birth and that pessary can reduce the PROM.

Preeclampsia

Preeclampsia is a strong factor that can lead to preterm birth (OR=4.43) [33]. Frequent monitoring of blood pressure is crucial for all pregnant women, particularly those with a history of hypertension. The target blood pressure should ideally be less than 130 mmHg. Utilizing various blood-pressure-lowering agents can help achieve this target. Lowering blood pressure to less than 130 mmHg has been associated with a one-third reduction in the risk of severe hypertension. Moreover, it contributes to a decrease in the incidence of preeclampsia, severe preeclampsia, placental abruption, and preterm birth [34].

Women at high risk of preeclampsia can be classified into high-risk: those with one or more of the following conditions: a history of preeclampsia, chronic hypertension, multiple gestation, diabetes mellitus, autoimmune diseases, or kidney diseases; and moderate risk: those with more than one of the following factors: BMI >30, first pregnancy, age >35 years, family history of preeclampsia, personal history factors, or sociodemographic factors [35].

Pravastatin (20 mg bid) can be used to prevent preeclampsia and preterm birth in high-risk women; compared to control, pravastatin reduced preterm birth (16.1 vs. 36%, p=0.003, OR=0.340), and was generally related to better perinatal outcomes [36].

Vaginal progesterone 400 mg twice daily when given in the first trimester of pregnancy can prevent preeclampsia and hypertensive diseases of pregnancy, while when initiated in the second or third trimester, it has no value in this matter [37].

Antiplatelets prophylaxis

Low-dose antiplatelets such as aspirin can be used to prevent preeclampsia, as it is caused by increased levels of the vasoconstrictor agent thromboxane and decreased production of vasodilator agents such as prostacyclin. Low-dose aspirin prophylaxis in nulliparous low-risk women with singleton pregnancy didn’t cause a significant decrease, neither in preeclampsia nor in other hypertensive disorders; it decreased the risk of preterm birth <34 weeks significantly (RR=0.50, p=0.04) and was small for gestational age, with no increase in the risk of antepartum or postpartum bleeding [38].

The use of low-dose aspirin in the prevention of superimposed preeclampsia in women with chronic hypertension has no significant role in decreasing the risk of preterm preeclampsia or superimposed preeclampsia, even with early initiation of the drug. Richards et al. (2023) [39] also showed a significant decrease in preterm birth (OR, 0.63; moderate-quality evidence). There was no effect on perinatal mortality or the small gestational age [39], while Man et al. (2021) [38] and Duley et al. (2019) [40] declared the effectiveness of low-dose aspirin.

Different results were provided by Duley et al. (2019) [40], who concluded from data from 60 trials and 36716 women that antiplatelets can decrease the risk of proteinuria preeclampsia (RR=0.82; high-quality evidence) and that they can lead to postpartum hemorrhage >500 ml (moderate-quality evidence). Data from 47 trials showed a slight reduction in preterm birth <37 weeks (RR=0.91; high-quality evidence).

Out of nine studies included in Duley et al. (2019) [40] and 10 studies in Man et al. (2021) [38], only two randomized controlled trials in each of them used doses ≥100mg, and the rest of the studies used doses lower than 100 mg. The United States Preventive Services Task Force recommended the use of a daily dose of 81 mg/d aspirin as a prophylaxis after the 12th week to the 28th in high-risk individuals, ideally before the 16th week of gestation continued until delivery [41].

Diabetes mellitus and lifestyle

Gestational diabetes mellitus (GDM) is one of the risk factors for preterm death, so prevention of it will lead to a decrease in many unwanted complications. Although lifestyle modifications such as diet, health education, and management of weight in patients at risk of GDM significantly reduced the risk of GDM by 46.9% and pregnancy-induced hypertension by 74.2%, they did not affect the risk of preterm birth or macrosomia [42].

Lifestyle modifications such as diet and physical activity can reduce gestational weight gain and may even reduce postnatal weight retention, GDM, hypertension, cesarean section, preeclampsia, and preterm birth [43]. Women with a high triglyceride-glucose index may be at increased risk for developing GDM; dietary fibers can reduce the risk of GDM significantly, decrease preterm birth, and significantly increase gestational age [44]. Smoking is associated with adverse neonatal outcomes; when compared with women who stopped smoking during pregnancy, women who continued smoking had a significantly higher risk of low birth weight and preterm birth (aOR=1.31) [45].

Omega-3

Long-chain omega-3 fatty acids are essential fatty acids that can be obtained from dietary marine sources. It is sought to be linked to prolonged pregnancy, and its deficiency is linked to preterm birth. Omega-3 and omega-6 give prostaglandins and other oxylipins, which have an important role in labor initiation in normal pregnancy and also in pathological settings such as preterm labor.

Normally, there is a balance between the metabolites of omega-6, which are produced by uteroplacental units, and those produced by omega-3 locally. This balance maintains normal gestation and may be responsible for cervical ripening and the initiation of pregnancy. This process can be accelerated by increasing omega-6 metabolites or decreasing omega-3 metabolites, leading to preterm birth [46].

Therefore, screening for omega-3 levels at early pregnancy and administering needed supplementation. The recommended omega-3 long-chain polyunsaturated fatty acid supplementation for women with low levels is to be a total of 1000 mg of docosahexaenoic acid and eicosapentaenoic acid, and it should begin before the 20th week of gestation. And women who have normal levels should maintain their omega-3 intake [47].

Bed rest

Although bed rest has always been the first step in management for women who are at risk, there is no scientific evidence to support or contradict describing bed rest, neither in singleton [48] nor multiple pregnancies [49].

Prophylactic antibiotics

According to a meta-analysis, prophylactic antibiotics demonstrated benefits exclusively for women with a history of premature delivery who currently have bacterial vaginosis. However, there was no discernible value for patients with a history of preterm birth but without bacterial vaginosis, as prophylactic antibiotics did not show efficacy in preventing either preterm delivery or premature rupture of membranes [50]. The utilization of clindamycin to prevent spontaneous very preterm birth or late miscarriage in women with bacterial vaginosis, irrespective of their risk level, did not yield any discernible benefits when compared to a placebo [51].

## Conclusions

Preterm birth is a major cause of mortality. We can prevent it by modulating many of the causes, including the use of prophylactic low-dose antiplatelets, cerclage, maintaining normal blood pressure, continuous monitoring, regular ultrasound assessment, quitting smoking, alcohol, and any drugs, a healthy high-fiber diet, regular physical activity, and most importantly, using progesterone. Other interventions should be given more consideration and need more studies to determine whether to continue using them, such as bed rest, which may be a financial waste, or prophylactic antibiotic use.
